# Supplementary figures and images for: Translational lipidomics reveals BMP and its precursor LPG as biomarkers for CLN5 Batten disease
Source: bioRxiv. 2026 Mar 21:2026.03.19.712969. Preprint. [Version 1] doi: 10.64898/2026.03.19.712969 (PMC13015511; doi:10.64898/2026.03.19.712969)

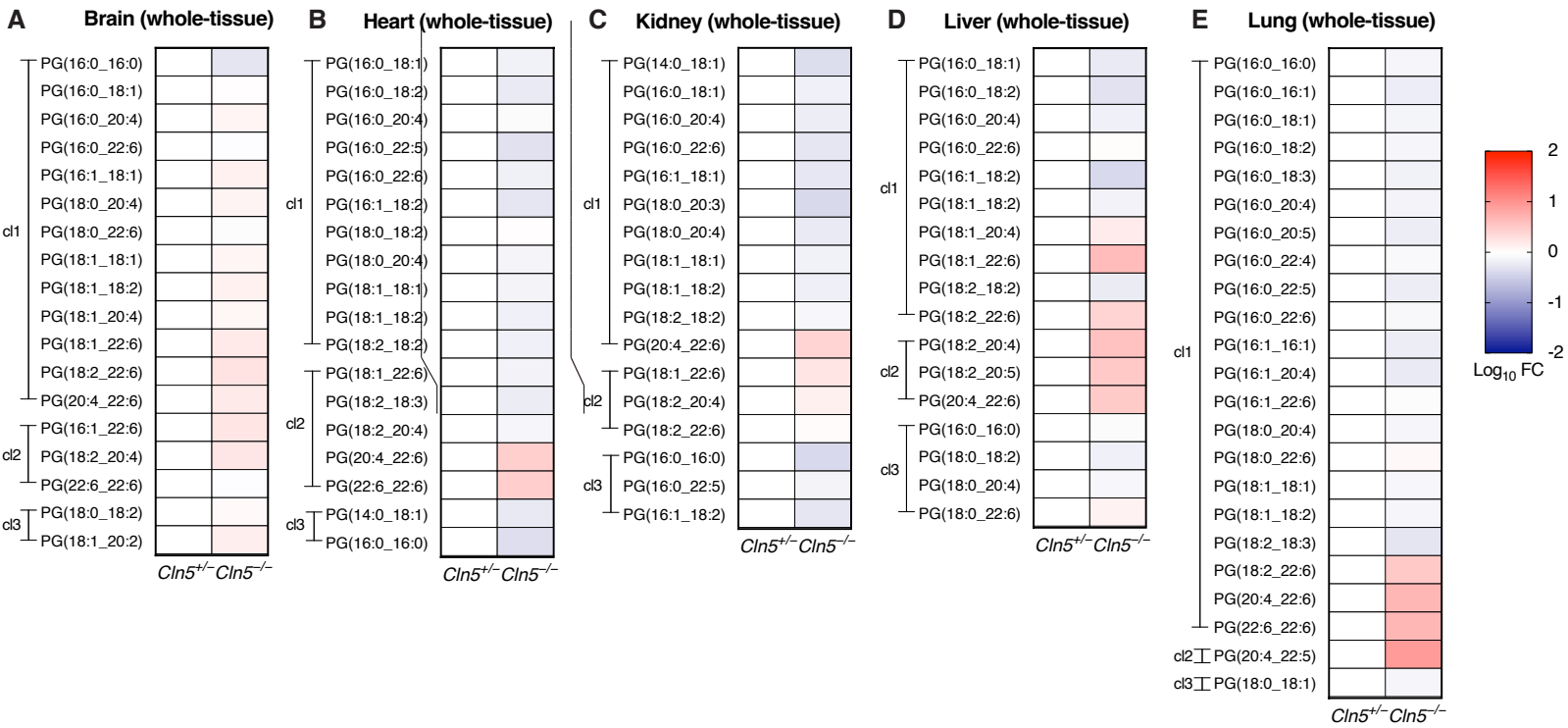

Supplement: Supplement 1 — Figure S1. Cln5−/− mice exhibit minimal change of PGs across tissues and in lysosomes. A-E. Targeted quantitation heatmaps of PGs from untargeted whole-tissue lipidomics, corresponding to Fig. 1A–E volcano plots: brain (A), heart (B), kidney (C), liver (D), and lung (E). Cln5−/− mice n = 5, Cln5+/− mice n = 10 for A-D, Cln5+/− mice n = 9 for E. F-G. Targeted quantitation heatmaps of PGs from untargeted LysoIP lipidomics, corresponding to Fig. 2A–B volcano plots: brain (F) and liver (G). Cln5+/− mice n = 4, Cln5−/− mice n = 3. Data from untargeted Orbitrap LC/MS. Fold change (FC) by ratio of means. p-values by Student’s t-tests. Targeted quantitation heatmaps normalized by PE(18:1_18:1). All quantified species in heatmaps were manually and rigorously validated for proper annotation and integration. PGs with confidence level 1 (cl1) label are annotated by definitive +NH4 MS2 and −H MS2. PGs with cl2 label are annotated by −H MS2 and relative retention time (RT) to an isomeric cl1 BMP in the same tissue. PGs with cl3 label are annotated by −H MS2 and relative RT to an isomeric cl1 BMP in a different tissue. [file media-1.pdf]

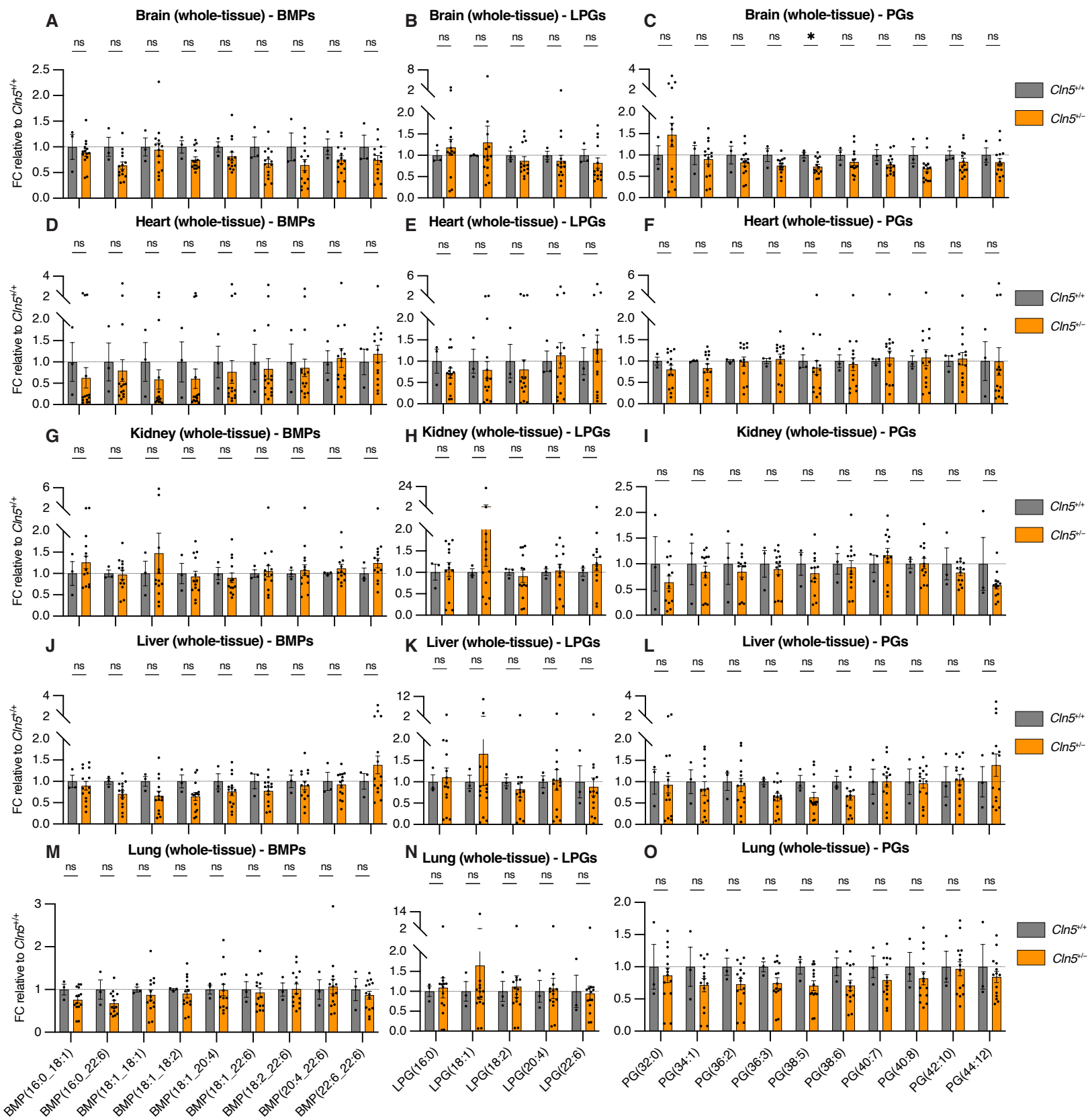

Supplement: Supplement 2 — Figure S2. Cln5+/+ and Cln5+/− mice exhibit no difference in BMPs, LPGs, and PGs. A-O. Targeted quantitation heatmaps of BMPs, LPGs, and PGs from targeted whole-tissue lipidomics of Cln5+/+ and Cln5+/− mice: brain (A-C), heart (D-F), kidney (G-I), liver (J-L), and lung (M-O). Cln5+/+ mice n = 3, Cln5+/− mice n = 14 for A-F and J-O, Cln5+/− mice n = 13 for G-I. Data from targeted QQQ LC/MS. Fold change (FC) by ratio of means. Targeted quantitation heatmaps normalized by PC(18:1_18:1). p-values by Student’s t-tests. Targeted quantitation normalized by PC(18:1_18:1). Statistical comparisons are marked as: ns (not significant), * (p < 0.05). [file media-2.pdf]

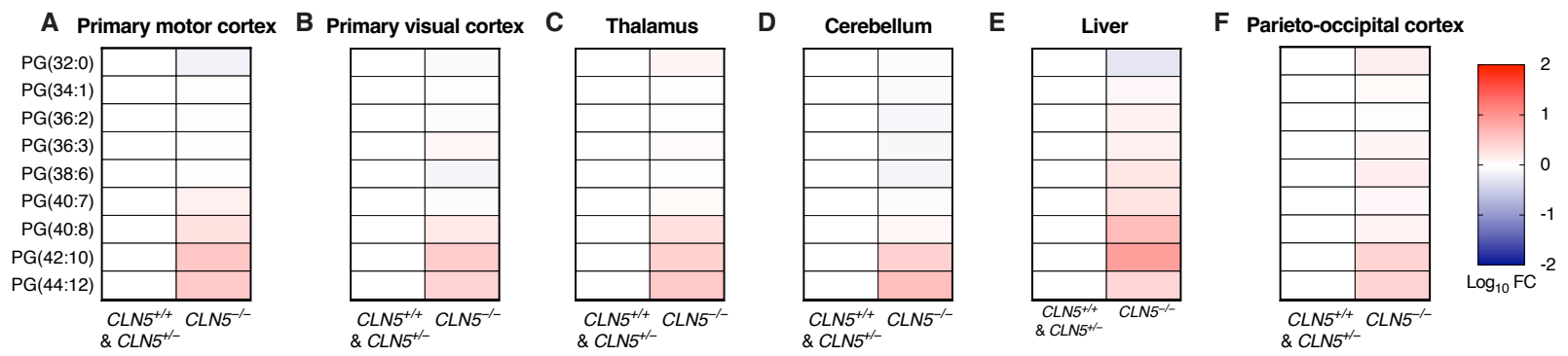

Supplement: Supplement 3 — Figure S3. CLN5−/− sheep exhibit minimal change of PGs across brain regions and liver. A-E. Targeted quantitation heatmaps of BMPs and LPGs from targeted whole-tissue lipidomics of CLN5+/+, CLN5+/−, and CLN5−/− sheep, corresponding to Fig. 4A–E heatmaps: primary motor cortex (A), primary visual cortex (B), thalamus (C), cerebellum (D), and liver (E). CLN5+/+ n = 1, CLN5+/− n = 2, CLN5−/− n = 3. CLN5+/+ and CLN5+/− have been grouped together for analysis as both animals present as healthy controls. F. Targeted quantitation heatmaps of BMPs and LPGs from targeted whole-tissue lipidomics of CLN5+/− and CLN5−/− sheep, corresponding to Fig. 4F heatmap: parieto-occipital cortex (F). CLN5+/− n = 4, CLN5−/− n = 3. Data from targeted QQQ LC/MS. Fold change (FC) by ratio of means. Targeted quantitation heatmaps normalized by PC(18:0_18:1). [file media-3.pdf]
